# Supplementary material for: Genotype specific pathogenicity of hepatitis E virus at the human maternal-fetal interface
Source: Nat Commun. 2018 Nov 12;9:4748. doi: 10.1038/s41467-018-07200-2 (PMC6232144; doi:10.1038/s41467-018-07200-2)
Supplement: Supplementary file 1 — Supplementary Information [file 41467_2018_7200_MOESM1_ESM.pdf]

Genotype Specific Pathogenicity of Hepatitis E Virus at the Human Maternal-Fetal Interface

Jordi Gouilly[1], Qian Chen[1], Johan Siewiera[2], Géraldine Cartron[3], Claude Levy[4], Martine Dubois[5], Reem Al-Daccak[6], Jacques Izopet[1,5], Nabila Jabrane-Ferrat[1,*], and Hicham El Costa[1,5,*]

[1]Centre of Pathophysiology Toulouse Purpan, INSERM U1043, CNRS UMR5282, Toulouse III University, 31024 Toulouse, France.
[2]University of California San Francisco, School of Medicine, Laboratory of Medicine, San Francisco, California, USA.
[3]Service de Gynécologie-Obstétrique, Hôpital Paule de Viguier, Centre Hospitalier Universitaire, 31059 Toulouse, France.
[4]Service de Gynécologie-Obstétrique, Clinique Sarrus-Teinturiers, 31300 Toulouse, France.
[5]Laboratoire de Virologie, Institute of Federative Biology, Centre Hospitalier Universitaire, 31059 Toulouse, France.
[6]INSERM UMRS976, Université Paris Diderot, Hôpital Saint-Louis, 75010 Paris, France.

[*]These authors contributed equally and share senior authorship.

Supplementary information

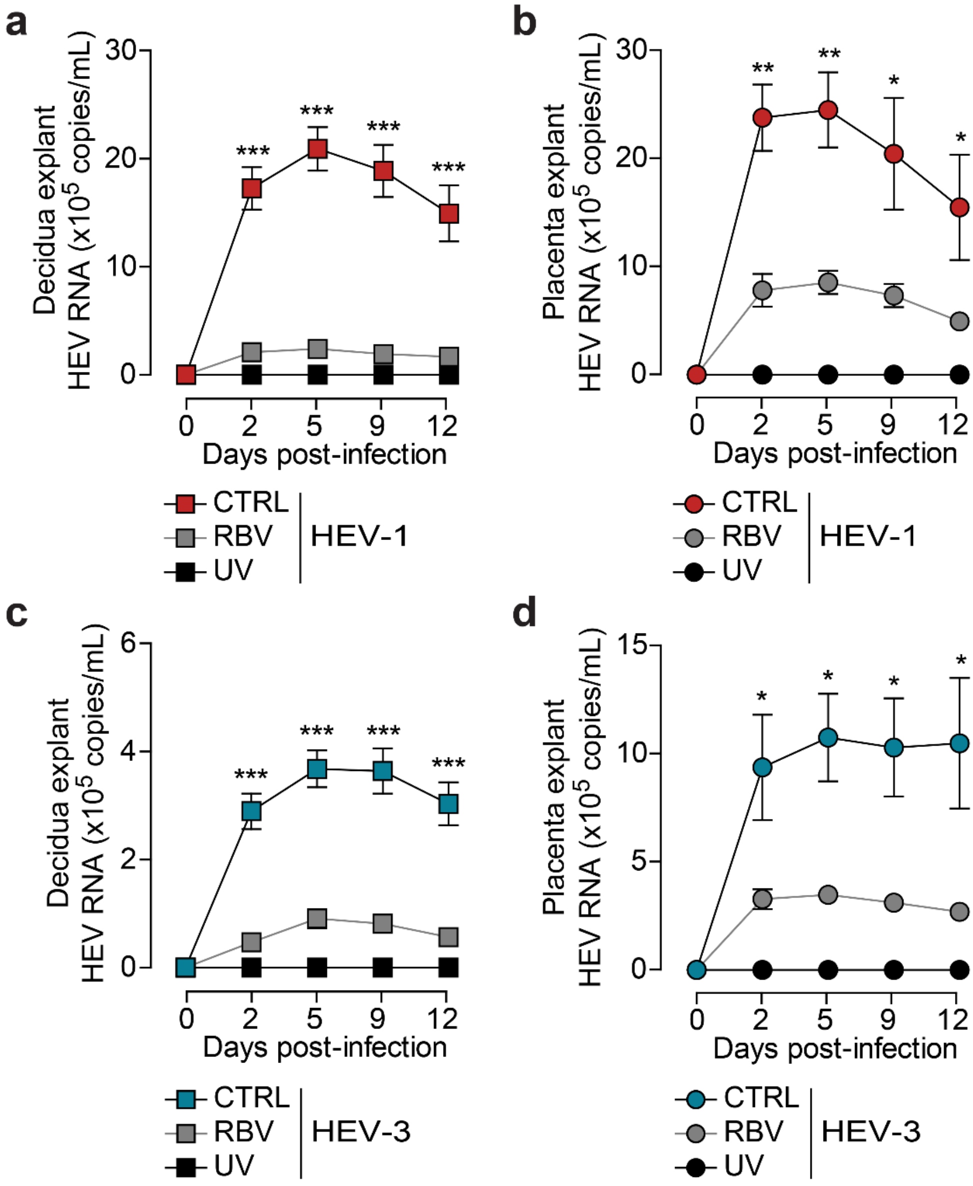

Supplementary Figure 1. Ribavirin inhibits HEV-1 and HEV-3 replication in decidual and placental tissues. Kinetics of HEV virus production from explants established from the decidua (**a**,**c**) and placenta (**b**,**d**), infected with HEV-1 (**a**,**b**; in red), or HEV-3 (**c**,**d**; in cyan). After infection, tissues were left untreated (CTRL) or treated with 50µM of ribavirin (RBV, in grey). UV-irradiated virions failed to infect decidual and placental explants (UV, in black). RNA levels were then measured in tissue culture supernatants by RT-qPCR. Data represent means values ± S.E.M. of three independent donors. (*) denotes a statistical comparison between CTRL- and RBV-treated infected tissues. * P<0.05; ** P<0.01; *** P<0.001 by two-way ANOVA with Bonferroni post-hoc test.

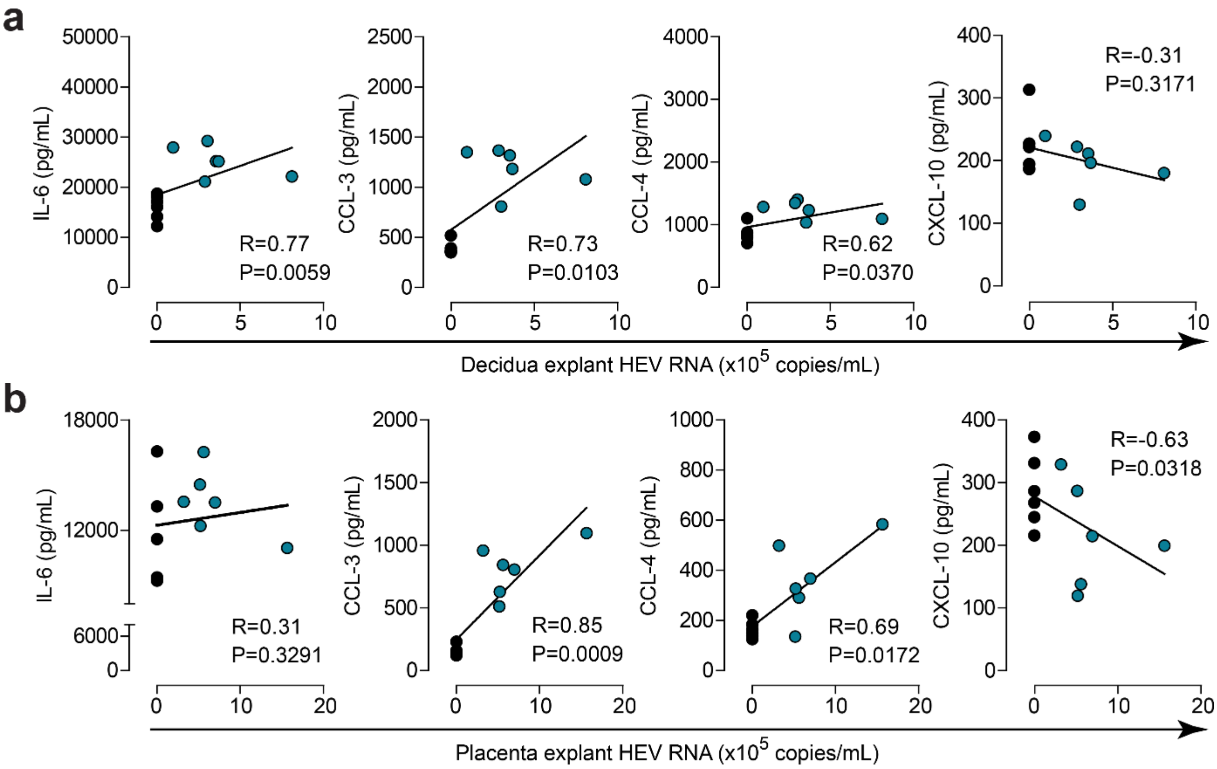

Supplementary Figure 2. HEV-3 infection of decidual and placental tissues skews their secretory function. (**a,b**) Correlation between IL-6, CCL-3, CCL-4 or CXCL10 secretion and viral production in the decidua (**a**) and placenta (**b**) two days after HEV-3 infection. Black and cyan points represent mock-, and HEV-3-infected tissues respectively. The Spearman's rank correlation test P value and R coefficient are indicated in each graph. Data represent values of six independent donors.

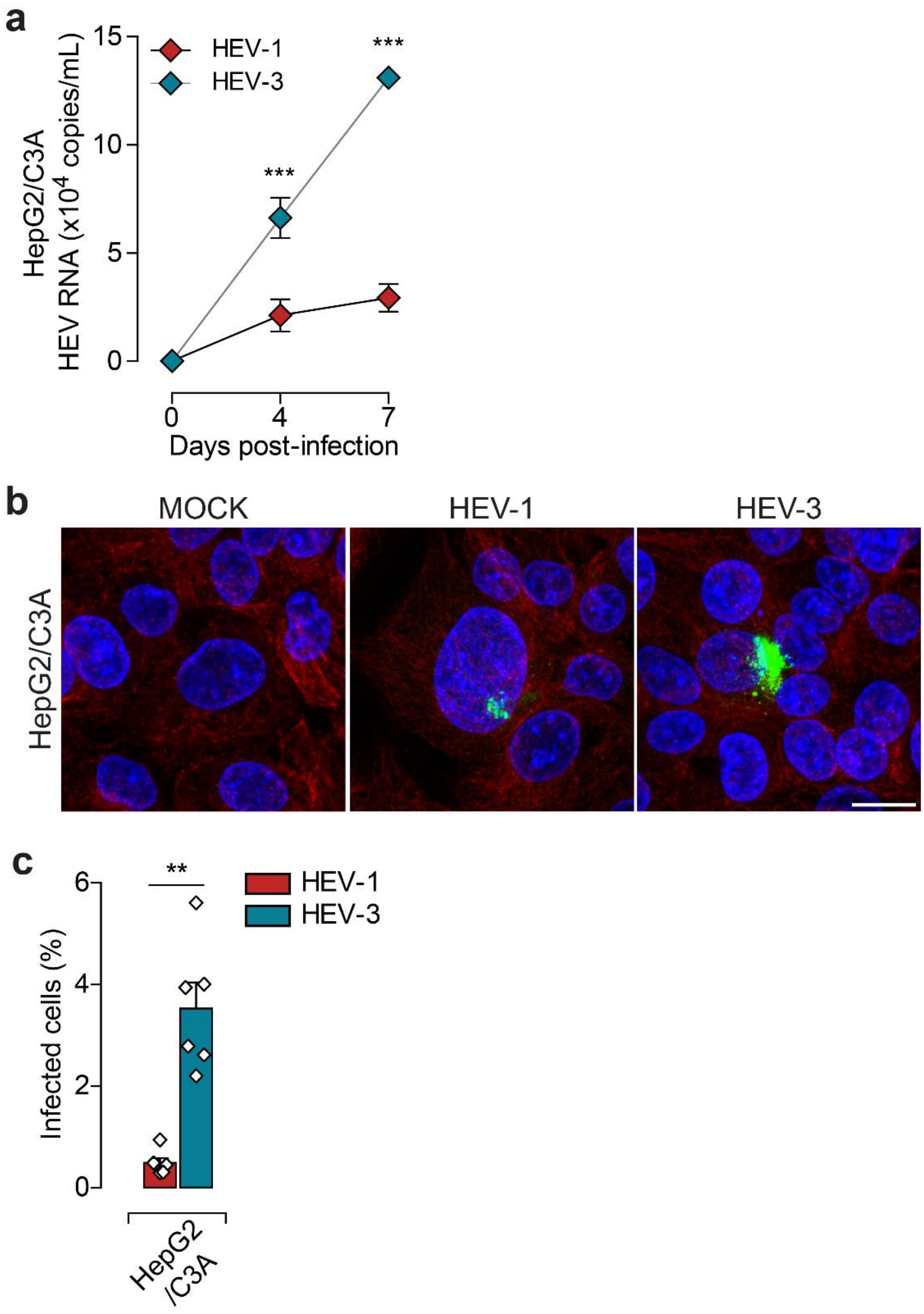

Supplementary Figure 3. HepG2/C3A cells are targets of HEV-3 and, to a lower extent, HEV-1 infection. (**a**) Kinetic of viral RNA production in HepG2/C3A cell line, infected with either HEV-1 (in red) or HEV-3 (in cyan). Virus production was determined by RT-qPCR in culture supernatants. (**b**) Representative images of HepG2/C3A cells seven days after mock, HEV-1, or HEV-3 infection. Maximum intensity projections are shown. Staining indicates the ORF2 viral capsid protein (green), α-tubulin (red), and nuclei (blue). Scale bar, 20µm. (**d**) Bar graph illustrating the percentage of infected HepG2/C3A cells seven days after HEV-1 (red) or HEV-3 (cyan) infection and determined by ORF2 staining. Data represent mean values ± S.E.M. of three independent experiments. (*) denotes a statistical comparison made between HEV-1 and HEV-3 infected cells. ** $P<0.01$; *** $P<0.001$ by two-way ANOVA with Bonferroni post-hoc test (**a**) and paired t-test (**c**).
